# Effectiveness of acute myocardial infarction interventions on selected outcomes among community dwelling-older adults: a systematic review and meta-analysis

**DOI:** 10.1038/s41598-023-45695-y

**Published:** 2023-10-28

**Authors:** Samoraphop Banharak, Alin Metprommarat, Wiriya Mahikul, Thanakrit Jeamjitvibool, Anusorn Karaket

**Affiliations:** 1 Department of Gerontological Nursing, Faculty of Nursing, Khon Kaen University, Khon Kaen, Thailand; 2 Queen Sirikrit Heart Center of the Northeast, Khon Kaen, Thailand; 3 Princess Srisavangavadhana College of Medicine, Chulabhorn Royal Academy, Bangkok, Thailand; 4 Princess Agrarajakumari College of Nursing, Chulabhorn Royal Academy, Bangkok, Thailand; 5 Nursing Department, Rasisalai Hospital, Sisaket Province, Thailand

**Keywords:** Cardiology, Health care, Risk factors, Signs and symptoms

## Abstract

The older adult is an influential group experiencing acute myocardial infarction, delaying treatment and causing a high mortality rate. Factors related to their delay differ from other age groups, and their specific characteristics are barriers to recognizing their symptoms and learning new information. Therefore, specific innovative methods related to their limitations and needs should be considered when developing interventions promoting on-time treatment. This study aims to review intervention details and their effects on knowledge, belief, decision-making, rate of calling 911, and mortality among community-dwelling older adults at risk or after a first myocardial infarction compared to receiving usual care or no intervention. The 12 databases were searched unlimitedly until July 30, 2022. The two researchers independently reviewed the articles, and the third reviewer broke the tight when disagreement was found. Data were extracted, kinds of interventions were grouped, and intervention details were summarized narratively. Finally, the selected outcomes were analyzed by meta-analysis using a fixed and a random-effects model. Eleven articles were for final review. Interventions were categorized into eight groups: direct mail, community-based, multi-group health education, innovation methods, tailored education, structured education, tricked intervention promoting memory and concern, and nurse-based case management. Finally, the meta-analysis found that only innovative methods could increase the rate of calling 911 and taking aspirin (Odd ratio = 2.55; 95% CI = 1.01-6.44). In contrast, there were no statistically significant differences in the rate of affecting time to first unplanned readmission or death and time delay to the emergency room. Results recommended that effective and specific interventions must be developed and strengthened to promote older adults surviving acute myocardial infarction.

**Clinical Trial Registration Number:** PROSPERO CRD42021247136.

## Introduction

The world population nowadays lives longer; their life expectancy is sixty and older^1^. Unfortunately, their extended lifetime is with functional decline and chronic diseases that cause their illness to become more complicated and hinder them from being detected and treated^2,3^. Reduced body reserve, Atypical presentation, Multiple pathologies, Polypharmacy, and Social adversity (RAMPS) can describe why it is complicated when providing care and treatment among older adults^4^. Reduced body reserve led to older persons' health conditions quickly deteriorating and their ability to wait less than other age groups because of lower energy reservation. Atypical presentation confuses older adults, their family members, and healthcare staff about the origin of problems, causing missed or delayed diagnosis and treatment. Multipatology causes them to use Poly medicine, and both join in making signs and symptoms more complicated. Finally, older adults need help from their family members and support from others in their community for emergency and long-term care situations, which is defined as Social adversity^3^. The five clinical features complicate older adults’ diagnosis, care, and treatment. It is a serious problem if older adults confuse their symptoms and delay treatment in an emergency, especially acute myocardial infarction (AMI), which needs quick and accurate responses within two hours, defined as the golden period^2^. The specific intervention aware of older adults’ limitations and needs to promote quick and accurate decisions and prevent treatment delay should be considered.

## Background

AMI occurs when the blood flow that brings oxygen to the heart muscle is immediately and severely reduced or cut off because of plague rupture or acute vasoconstriction, causing an emergency^2^. AMI is a time-related recovery disease called time-is-muscle since the myocardial damage or recovery depends on the time the patients decide and receive treatment; however, delayed decision and high mortality rates are still found^2^. Studies reported that the patient with a longer interval between the onset of symptoms and treatment is likelier to experience complications and die than the patient who did not delay treatment^5,6^. This serious health problem is one of the leading causes of death in the developed world, with prevalence approaching three million people worldwide^7^. About every 40 s, someone in the United States has an AMI, and more than one million deaths in the United States annually^2^. Based on the National Health and Nutrition Examination Survey 2017 to March 2020 data, the prevalence of cardiovascular diseases in adults in the United States was 48.6% overall (about 127.9 million), which increases with age, and older adults substantially contribute to the mortality caused by cardiovascular morbidity^8^. In the same year, mortality data, cardiovascular diseases currently claim more lives each year; about 207 of 100,000 people died, and the leading cause is AMI and stroke^2^. According to data from 2005 to 2014 from the Atherosclerosis Risk in Communities Study, the estimated annual incidence of myocardial infarction is 605,000 new attacks and 200,000 recurrent attacks. Both cause cardiogenic shock and cardiac arrest, becoming the major cause of death^2^.

Around 5% of patients with AMI develop cardiogenic shock with a mortality of 40–50%, found outside the hospital while seeking treatment. The morbidity and mortality associated with AMI are proportionate to the time to receive treatment from the onset of symptoms^9^. Another study also reported that laypeople in the United States initiated cardiopulmonary resuscitation in 40.2% of out-of-hospital cardiac arrests in 2021^10^. In the United States, sudden cardiac arrest and experience worse survival after sudden cardiac arrest were still high^2^. Every year, more than 356,000 Americans experience an out-of-hospital cardiac arrest, and 60 to 80 percent die before arriving at the hospital, and this is often found among females and older adults^2,8^. The atypical presentation often found among female and older adults, confusing them to decide to get treatment lately, might be the origin of the problem^7^.

Mortality associated with AMI is directly linked to the time to receive treatment and missed diagnoses^9^. Then, delayed decisions and treatment are areas of concern. Based on the systematic review, Huriani et al. reported that the mean time from symptom onset to first medical contact was 12.7 h, ranging from 10 min to 96 h. Older, female, illiterate, living in a rural area, and financially limited were associated with longer treatment delays. The lack of a developed emergency transportation system and poor communication and organization between community and interventional facilities were also cited as significant contributors to treatment delays^11^. Another cause of delay is the atypical presentation of AMI among older adults, which confuses them during their symptom recognition and decision-making^9^. American Heart Association reported that 805,000 patients experienced first and recurrent events^2^; however, about 170,000 are silent, and over 50% experience atypical symptoms of AMI^8,9,12–14^. The atypical presentation was found in over 50% of older adults, and two-thirds delayed the decision to get treatment^9,12,14^. Moreover, over half of this group are older adults who died before arriving at the hospital and have no chance to receive treatment, although AMI treatments are very effective^12,14^. Health professionals are aware of typical AMI presentation; however, atypical AMI is difficult to diagnose and confuses older adults, their family members, and healthcare staff. On the other hand, it is likely to impact delayed decision-making and cause high morbidity and mortality.

Khan et al. summarized that atypical presentations of myocardial infarction are vast; patients may have chest pain without typical characteristics of angina pectoris or may not have chest pain. Most patients were older adults and commonly presented with pain and discomfort in the abdomen, head, and neck regions^9^. The other two studies reported that atypical clinical features, such as neck pain, pain in the back, throat pain, ear discomfort, and hiccups, are not uncommon. Craniofacial pain can be the sole symptom in up to 6% of patients with AMI. Women and older adults need special mention as they often present with atypical symptoms, and a high index of suspicion is required^9,12^. A patient who is 50 years or older, having comorbidities such as diabetes, hypertension, dyslipidemia, history of tobacco or marijuana usage, and presenting with prodromal symptoms like shortness of breath, dizziness, fatigue, syncope, gastrointestinal discomfort or head/neck pain should be suspected for atypical AMI^9^. This atypical symptom can lead to delayed decision-making and diagnosis, suboptimal treatment, and detrimental outcomes. To avoid such mishaps, accurate and timely interpretation of atypical clinical symptomatology of AMI has a vital bearing on patient triage, treatment, and subsequent management^9^.

Beyond worsening health conditions and high mortality rates, AMI made up the cost of treatment for both direct and indirect costs. According to Healthcare Cost and Utilization Project data from the Agency for Healthcare Research and Quality for 2018, 481,780 percutaneous coronary interventions were performed in patients in the United States^2^. The average annual direct and indirect cost related to AMI in the United States was an estimated $407.3 billion from 2018 to 2019. The estimated direct costs related to AMI in the United States increased from $103.5 billion in 1996 to 1997 to $251.4 billion from 2018 to 2019. By event type, hospital inpatient stays accounted for the highest direct cost ($111.4 billion) from 2018 to 2019 in the United States^8^. If they delay treatment, they may experience complications, causing longer lengths of stay and higher treatment costs. All of the above reflect that AMI is an emergency and critical situation that needs appropriate and quick response to receive treatment on time to decrease worsening health conditions, mortality rate, and cost of treatment. This condition is associated with age and causes a high mortality rate for this group of people, especially those who died before arriving at the hospital and had no chance to get treatment. Then, delayed decision-making is an area of concern^2^.

In conclusion, older adult is a significant group of people who experience AMI and delay seeking treatment. The results from this situation are that most of this vulnerable group of people delayed seeking treatment and died before arriving at the hospital. Moreover, older adults who receive treatment late have high rates of long length-of-stay, high cost of treatment, in-hospital mortality, and mortality of 30 days after discharge^9,11^. Specific interventions to promote decision-making and prevent delay in seeking treatment among older adults from the literature review are needed to deal with this emergency and critical situation. The interventions to promote quick and accurate decisions to receive treatment on time are an area of concern. From an initial review, we found that several systematic reviews provided various pathways, techniques, materials, and contents of interventions to deal with this critical problem^11,15,16^. Unfortunately, these interventions are for all age groups of AMI patients; however, factors causing older adults to delay treatment differ from other age groups, especially atypical symptoms, knowledge, belief, and their functional decline related to aging. The specific interventions that promote decision-making and prevent delaying treatment for older adults are not promptly synthesized. This review was conducted to summarize all details and types of interventions to promote decision-making, prevent delaying treatment for older adults, and explore their effectiveness.

## Aims

This study aimed first to review the components and details of AMI interventions to promote decision-making and prevent delaying treatment. Then, interventions’ details were summarized and guided for intervention development for a specific group of community-dwelling older adults. Secondly, the effects of the interventions on selected health outcomes among community-dwelling older adults were also explored and demonstrated.

## Methods

### Design

A systematic review with a narrative summary of interventions' details and a meta-analysis of selected health outcomes was undertaken. The Preferred Reporting Items of Systematic Reviews and Meta-Analysis (PRISMA) checklist guidelines and the standardized critical appraisal instruments from the Joanna Briggs Institute^17^ were used to conduct this review. The protocol to conduct this systematic review was prospectively registered and published with PROSPERO (CRD42021247136).

### Search strategies

Keywords were identified for searching by using PICO as population, intervention, comparison, and outcomes. The population is community-dwelling older adults at risk or after a first myocardial infarction. The intervention is AMI interventions, the comparison is no intervention or usual care, and the outcome is decision-making and on-time treatment. The search terms for population included “older adult,” “older people,” “elderly,” “aging,” “senior citizen,” “chest pain,” “acute myocardial infarction,” “heart attack,” “acute coronary syndrome,” and “cardiac event.” The search terms for interventions are “health education,” “health literacy,” “community-based program,” “telehealth,” and “avatar health application.” The comparison search terms are “home visit” and “routine suggestion.” Finally, the search terms for outcomes are “knowledge,” “attitude,” “decision,” “time of delay,” “health-seeking,” “delayed time,” “pre-hospital delay,” “timely treatment,” and “seeking treatment.” The researchers used “OR” to connect wordings within the concept. However, “AND” was used to connect wordings between concepts. The search statement was developed and published in PROSPERO so another independent reviewer could duplicate and check.

Databases were unlimitedly searched for empirical articles up to July 30, 2022. The search was conducted in 12 databases: PubMed, CINAHL, SCOPUS, OVID, CENTRAL, ISI, ProQuest, ClinicalTrail.gov, Open grey, ThaiList, ThaiJO, and E-THESIS, using keywords.

### Inclusion and exclusion criteria

The inclusion criteria included the studies (1) at least 80% of participants aged 60 or older, (2) were randomized controlled trials (RCT) or quasi-experimental studies and achieved 60% of scores from each critical appraisal tool, such as 8 out of 13 or 6 out of 9 from the Critical Appraisal for RCT and quasi-experimental studies, respectively, (3) reported components and details of the AMI interventions to promote decision-making and prevent delaying treatment, (4) reported relevant statistical results of the AMI interventions to promote decision-making and prevent delaying treatment, including knowledge, belief/attitude, calling 911 or EMS, mortality, readmission, appropriate action/taking aspirin, coping behavior/anxiety, awareness, time of delay/seeking, and malnutrition risk. Only three studies reported the quality of instruments, and (5) were published in English or Thai in both peer-reviewed journals or were thesis/dissertations. However, the study included hospitalized older adults, and the study included older adults with mild cognitive impairment or depression were excluded.

### Critical appraisal

Evidence levels were indicated using the hierarchical evidence pyramid from the JBI^18^. Before including the research articles, the selected studies' quality was assessed using the critical appraisal tool from JBI; the Checklists for RCT and Quasi-Experimental Studies were applied for this review^19,20^. Moreover, grading was provided for each study, which was included in the table of result reports^21^.

### Risk of bias

The selected studies were required to meet a positive response (i.e., “yes”) on a minimum of six out of nine for quasi-experimental studies and eight out of 13 for RCT^19,20^. Two reviewers independently assessed the risk-of-bias, and a risk-of-bias table was designed for each eligible study. Disagreements between review authors were resolved by mutual consensus and the third reviewer. Methodological quality was categorized into very low, low, moderate, and high-quality categories^21^. Critical appraisal results were also reported in narrative form and a table. All selected studies were reported their methodological quality and underwent data extraction and synthesis.

### Study selection and data extraction

This process included two steps. First is a study selection. The two reviewers conducted the study selection independently for inclusion, with their decisions blinded. This process was completed in two stages, initially based on titles and abstracts screening and then by reviewing the full text of the articles retained in the first step. We resolved any disagreements regarding the selection of studies by consensus. The two reviewers recorded any decisions in reference management software, Rayyan reference management. This step yielded a PRISMA Flow Chart after the screening process.

The second step is data extraction. Before starting this process, the codebook and data extraction forms were developed. The characteristics of each included study were extracted using a data extraction form, which included (1) the studies’ authors, (2) study designs, (3) settings, (4) participants, (5) levels of evidence certainty and methodological quality, (6) components of the AMI interventions; (7) times of outcomes measuring; and (8) the statistical results for selected outcomes of the AMI interventions. A codebook was revised after pilot testing with five studies. After that, two reviewers conducted data extraction independently, and disagreements between the reviewers were solved through mutual discussion. We attempted to contact included study investigators for unreported data or additional details needed for the meta-analysis generation; four studies were found, and the principal study investigators were contacted. Finally, we received all the information and statistics needed.

## Data analysis

### Qualitative results

The contents and details of programs or interventions were extracted and synthesized to support the first aim. The narrative methods were applied for this section. Moreover, themes were set to categorize groups of interventions. Essential and specific details included in the AMI interventions from selected studies were provided under each theme. These details help guide the effective intervention for practice and future study. Finally, some of the selected outcomes were impossible to include for meta-analysis due to the heterogeneity of the study population, outcome measures, time of measuring, and data analysis across the studies; the p-values of these selected outcomes were provided and reported in the table.

### Quantitative results

We included studies reporting odds ratios (unadjusted or adjusted OR) of calling 911 and taking aspirin among AMI patients with and without intervention. Hazard ratios were used to conduct a pooled analysis of the association between people receiving an intervention and those without any intervention regarding affecting time to first unplanned readmission or death. The standardized mean differences were used to analyze the association between people receiving an intervention and those without any intervention regarding delay time to the ER. A meta-analysis was conducted only for categories with sufficient available data, typically requiring more than one study to combine the outcomes.

The DerSimonian and Laird based on the inverse-variance weighted average approach for meta‐analyses with random effects were applied if the study heterogeneity was high^22^. However, if necessary, the fixed-effect model with inverse-variance weights was also applied if the study heterogeneity was low. The supposed clinical or considerable statistical heterogeneity was present (Statistically significant when p-value < 0.10 using a chi-squared test (Cochran Q test) and I^2^ > 50%). In that case, the findings with a narrative approach following synthesis without meta-analysis guidelines were applied. Publication bias was visually assessed using Begg’s funnel plots and statistically assessed with Egger’s test. The analyses were conducted using statistical software of STATA version 16. Finally, results were reported following the PRISMA guideline for reporting systematic review and meta-analysis.

## Validity, reliability, and rigor

A research team from different disciplines and expertise conducted this study. The systematic review experts and librarians worked together to develop a search statement, select essential databases related to the study topic, and search for articles together. The team developed and proved the systematic review protocol and search statement. The principal researcher prospectively registered a study protocol before conducting this systematic review and strictly followed the protocol to reduce bias and increase validity and reliability. An independent review was provided for all processes. A third independent researcher provided a third opinion that helped break the tight when disagreement was found. Moreover, if primary researchers did not provide sufficient data for generating meta-analysis, we contacted the corresponding author via provided e-mails. Fortunately, we received all we needed from the corresponding authors. Finally, statisticians took part in data analysis, especially meta-analysis generation, to confirm the accuracy of data analysis for this study.

## Results

**Figure 1:**
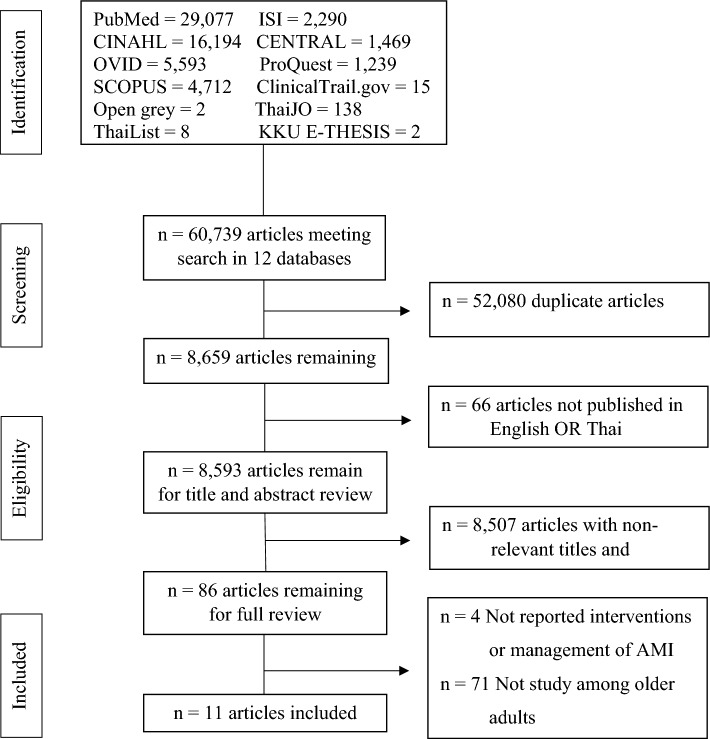
Flow chart of the review process and results.

The initial search found 60,739 articles from 12 databases, and 8,659 articles were the rest after duplicate. Based on the inclusion and exclusion criteria, 11 articles were selected for the final comprehensive review. The PRISMA flow diagram of the information flow during the review process is displayed in Fig. 1.

### Characteristics of included studies

Of 11 studies^23–33^, eight were conducted before 2012^23–30^. Nine studies were in the USA^23–31^, and two were in Germany^32,33^. Ten studies aimed to explore the effectiveness of intervention/health education on health outcomes^24–33^; however, one focused only on elevating the use of emergency services (call 911 or call for emergency medical service: EMS)^23^. The research designs were nine randomized controlled trials^23–27,29,31–33^ and two quasi-experimental studies^28,30^. The interventions included a direct mail intervention^23^, a community-based intervention program^24^, a Rapid Early Action for Coronary Treatment (REACT) trial^25^, three Heart Attack Survival Kit^23,27,29^, two structured/tailored educational/counseling interventions^28,30^, a Health education-Matters Of Your Heart using the acronym FACTSS & CURB^31^, and two nurse-based case management for older patients^32,33^. Five teams used multiple pathways^24,26,27,32,33^, including face-to-face/home visits, direct mail/telephone, and advertisement. The other five teams used only face-to-face/home visits^25,28–31^; another used only direct mail/telephone^23^. Eleven studies reported the selected outcomes based on the inclusion criteria, including knowledge, belief/attitude, calling 911 or EMS, mortality, readmission, appropriate action/taking aspirin, coping behavior/anxiety, awareness, time of delay/seeking, and malnutrition risk. Only three studies reported the quality of instruments (Table 1).

**Table 1:** Characteristics of included studies.

Authors: countries	Designs: aims	Interventions: ways of distributing intervention	Selected outcomes	Major condition of study	Settings	Participant’s age	Gender:	Ethnicity	Incomes
Meischke et al.^23^: USA	RCT: To increase the use of emergency medical services via 911 calls and to reduce pre-hospital delay for individuals experiencing AMI symptoms	The Direct mail intervention: Direct Mail/Telephone	- Calling 911 or EMS - Time of delay/seeking	Mixed conditions (AMI/CAD patients & Normal People)	Mixed setting (Hospital & Community)	≥ 80% ≥ 60 years with no report mean age and a standard deviation	Intervention group (n = 4,101) : male = 54.9 (2,251) , female = 46.1 (1,890) control group (n = 1,343) : male = 55.4 (744) , female = 44.6 (599)	White: 92.3% vs. 91.6%, African American 4.1% vs. 5.2%, Asian: 3.2% vs. 2.9%, American Indian: 0.1% vs. 0%, Hispanic: 0.4% vs. 0.2%	Intervention vs. Control groups < 20,000 = 35.4% vs. 31.0% 20,000–39,999 = 21.9% vs. 22.9% 40,000–49,000 = 20.2% vs. 19.2% 50,0000 = 22.6% vs. 26.9%
Hedges et al.^24^: USA	RCT: To determine the impact of a communicating educational intervention to reduce patient delay time	A community-based intervention program: Multiple Pathways (Face-to-Face/Home Visit & Advertisement)	- Mortality - Awareness	Risk People	Community	≥ 80% ≥ 60 years with no report mean age and a standard deviation	Intervention group male = 65.4 (1,104) female = 34.6 (589) control group male = 61.5(814) ,female = 38.5 (510)	Mixed	Not report
Luepker et al.^25^: USA	RCT: To reduce patient delay from symptom onset to hospital presentation and increase emergency medical service (EMS) use	The Rapid Early Action for Coronary Treatment (REACT) trial: Face-to-Face/Home Visit	- Calling 911 or EMS - Time of delay/seeking	Mixed conditions (AMI/CAD patients & Normal People)	Community	≥ 80% ≥ 60 years with no report mean age and a standard deviation	No report	Reference vs. Intervention groups Black: 8.7% vs. 8.7%, White: 76.7% vs. 78%, Hispanic: 11.6% vs. 12.1%, and (other): 4.9% vs. 3.2%	Median household income: reference group = 29,896 (18,396–39,264), intervention group = 27,056 (15,890–32,842)
Meischke et al.^26^: USA	RCT: To evaluate the effectiveness of the heart attack survival kit	The Heart Attack Survival Kit: Multiple Pathways (Face-to-Face/Home Visit & Direct Mail/Telephone)	- Calling 911 or EMS - Appropriate action/taking aspirin	Normal People	Mixed setting (Hospital & Community)	100% ≥ 60 years with mean age of 73 and a standard deviation (SD) of 6.4	Male: 51% (n = 360), female 49% (n = 345)	American Indian	Income < 20,000 = 19%, 20,000–40,000 = 34%, 40,000–60,000 = 15%, > 60,000 = 7%
Meischke et al.^27^: USA	RCT: To test an intervention designed to (a) call 911 and (b) take an aspirin in response to AMI symptoms	The Heart Attack Survival Kit: Multiple Pathways (Face-to-Face/Home Visit & Direct Mail/Telephone)	- Knowledge - Calling 911 or EMS - Appropriate action/taking aspirin	Risk People	Community	100% ≥ 60 years with mean age of 73 and a standard deviation (SD) 8.2	Intervention group: Male = 37%(65) ,female = 63%(111) Control group : male = 37%(54) ,female = 63%(93)	Intervention vs. Control groups: white 95% (167) vs. 97% (143) and other 5% (9) vs. 3% (4)	Intervention vs. Control groups < 24,000$ = 16% vs. 22%, 25,000–39,000$ = 14% vs. 28%, 40,000–54,999$ = 15% vs. 10%, > 55,000$ = 18% vs. 18%, (p = .003)
Lefler et al.^28^: USA	Quasi-Experimental: To improve the accuracy of the perceived risk of MI perception, and increase knowledge of MI symptoms	A structured/tailored educational/counseling intervention: Face-to-Face /Home Visit	- Knowledge - Belief/attitude	Risk People	Community	100% ≥ 60 years with no report mean age and a standard deviation	100% were female	85% (77) were Caucasian and 15% (13) others	Classified two groups < 30,000$ and = or more than 30,000 but did not report the results
Meischke et al.^29^: USA	RCT: To increase utilization of 911 and self-administration of aspirin for seniors experiencing chest pain	The Heart Attack Survival Kit: Face-to-Face/Home Visit	- Calling 911 or EMS - Appropriate action/taking aspirin	Risk People	Community	100% ≥ 60 years with mean age of 78 and no report standard deviation	The proportion of males (39% in control group and 42% in intervention group)	Not report	Not report
Tullmann et al.^30^: USA	RCT: To investigate the effectiveness of educational and counseling intervention for older adults	A structured/tailored educational/counseling intervention: Face-to-Face /Home Visit	- Knowledge - Belief/attitude - Calling 911 or EMS - Coping behavior/Anxiety	AMI/CAD patients	Community	100% ≥ 60 years with mean age of 74 and a standard deviation (SD) 6.0	Total : male = 48% (55) ,female = 52% (60), Control group : male = 51% (29), female = 49% (28) intervention group : male = 45%(26) ,female = 55% (32)	White n = 83 (72%), African-American = 8 (7%), Hispanic = 14 (12%), Native American = 8 (7%), Asian/Pacific Islander = 1(1%), Other = 1(1%)	< $15,000 = 45%, $15,000 – $30,000 = 28%, $30,001 – $45,000 = 7%, $45,001 – $60,000 = 8%, > $60,000 = 9%
Kalman et al.^31^: USA	Quasi-Experimental: To increase women's knowledge of female prodromal and myocardial infarction (Ml) symptoms and the appropriate response to these symptoms	Health education-Matters Of Your Heart-using acronym FACTSS & CURB: Face to Face/Home Visit	- Knowledge	Risk People	Community	≥ 80% ≥ 60 years with mean age of 68 and no report standard deviation	100% women	The majority of the sample was Caucasian n = 46(92%), African American n = 2(3.9%), Other n = 1(2%)	Not report
Meisinger et al.^32^: Germany	RCT: To evaluate the effects of nurse-based case management for elderly patients discharged after an AMI from a tertiary care hospital	Nurse-based case management for elderly patients: Multiple Pathways (Face-to-Face/Home Visit & Direct Mail/Telephone)	- Mortality - Readmission - Time of delay/seeking	AMI/CAD patients	Community	100% ≥ 60 years with mean age of 75.4 and a standard deviation (SD) 6.0	Total Male 62% (204), Intervention 62.7% (101)/Control 61.3% (103)	Mixed	Not report
Kirchberger et al.^33^: Germany	RCT: To evaluate the nurse-based case management for elderly patients discharged from the hospital after an AMI	Nurse-based case management for elderly patients: Multiple Pathways (Face-to-Face/Home Visit & Direct Mail/Telephone)	- Mortality - Readmission - Coping behavior/Anxiety - Malnutrition risk	AMI/CAD patients	Community	100% ≥ 60 years with mean age of 75.2 and a standard deviation (SD) 6.0	Total Male 62% (204), Intervention 62.7% (101)/Control 61.3% (103)	Mixed	Not report

The major conditions of the study were risk people and people with CAD/AMI—nine implemented interventions in the community^24,25,27–33^. Seven studies were conducted among 100% of older adults^26–30,32,33^, and four were mixed age group, of which over 80% was people aged 60 and older^23–25,31^. Eight studies included both sexes^23,24,26,27,29,30,32,33^, nine included mixed ethnicity^23–25,27,28,30–33^, and six reported income^23,25–28,30^. Their interventions were eight face-to-face/acronym/teaching^24–27,29,31–33^. Seven studies were conducted for longer than six months^23–25,27,29,32,33^. Nine studies were not prospectively registered protocols^23–31^. Intervention details included essential information to understand AMI, detect symptoms, and prevent delayed treatment; however, nine focused on overall symptoms or physical reactions^23–28,30,31,33^. Seven studies provided enough detailed interventions that researchers or readers could follow the intervention step by step for their practice^24,26,28–30,32,33^. Only three studies reported that they gave incentives to their participants^28,29,31^. Eight studies did not report activities for their control groups^23–27,29–31^. Six studies measured outcomes two times (pre-posttest)^25,28–33^. The number enrolled in each group ranged between 50 and 226,958 participants. Significant results were found in the knowledge of AMI^30^, AMI symptoms^26,28^, risk factors^28^, perception^28,30^, functional status^33^, hand grip^33^, aspirin taking^26,27,29^, and the number of calling 911^25–27,29^ (Table 2). Ten studies were level 1.c of evidence^23–30,32,33^, and critical appraisal ranked 8–12 for RCT (Table 3) and 6–9 for quasi-experimental study (Table 4). Finally, seven studies were graded as high level^23–26,29,30,32^ (Table 5).

**Table 2:** Details of acute myocardial infarction interventions.

Author	Kinds of Intervention: Protocol Registration	Duration of Intervention	Duplicable	Incentive	Controlled Group Intervention	Measurement Timeframe	Number of Enrolled in Each Group	Results	Level of evidence
Meischke et al.^23^	- Direct Mail - Not registering the protocol	10 months (6 brochures, one every two months)	No	No	No received brochure	A year of follow up	At start-intervention: 4,104, control: 1,343 For analysis- intervention: 4,101, control: 1,343	The percentage of persons calling 911 in the three intervention groups was somewhat higher than in the control group (information = 63.3%, emotional = 64.2%, social = 61.8%, and control = 60.4%, p > .05 respectively). The mean delay time between each intervention group and the control group was insignificant (information = 183 min, emotional = 167 min, social = 173 min, and control = 173 min, p = .017)	1.c
Hedges et al.^24^	Multi-forms - Face to Face/Acronym/Teaching - Video or Audiovisual - Telephone-based screening: TBS - Not registering the protocol	Telephone-based screening of the intervention and control community groups over 18 months	Yes	No	Not receive any interventions	18-month period by community intervention	n = 4,885 intervention group = 1,689, drop out = 936 control group = 1,324, drop out = 936	The difference between adjusted intervention and control trends for the in-hospital death rate was insignificant (OR 1.00; 95% CI = 0.36 to 2.77). The adjusted reperfusion rate within six hours of symptom onset was significantly greater for AMI patients transported via EMS personnel (36% vs. 24%; p < 0.0001). Adjusted rates for reperfusion therapy use at any time within the first 12 h after ED presentation were seen for the selected study population (44% vs. 33%; p < 0.0001)	1.c
Luepker et al.^25^	Multi-forms - Face to Face/Acronym/Teaching - Advertisement - Video or Audiovisual - Not registering the protocol	In the collection phase: 4 months based line was evaluated, followed by 18 months of intervention and concurrent evaluation	No	No	Not receive any interventions	4 months at the baseline and then 18 months after the intervention	Intervention group 112,659 and control group 114,299	Delay time decreased in intervention communities by − 4.7% per year (95% CI − 8.6% to − 0.6%), but the change did not differ significantly from that reference communities (− 6.8% per year, 95% CI − 14.5% to 1.6%, p = .54). EMS use increased significantly in intervention communities compared with reference communities, with a net effect of 20% (95% CI 7% to − 34%; p < .005). The fatality rate of the intervention communities decreased from 3.23% to 2.43%, and in the reference communities decreased from 2.66% to 1.78%. However, these decreases were not significantly different	1.c
Meischke et al.^26^	Multi-forms - Face to Face/Acronym/Teaching - Advertisement - Direct Mail - Telephone-based screening: TBS - Not registering the protocol	Contact during daytime (9 a.m.) over 2 weeks periods, then contact by phone 1 week and 6 weeks after intervention	Yes	No	Not receive any interventions	Assess daytime at 9 a.m. over 2 weeks. Then contact by phone in week 1 and week 6 after the intervention	Total = 1200 (control 400, Direct mail 400, and EMT-Delivery 400). For analysis, control 223, Direct mail 214, and EMT-Delivery 268	The intervention group reported a greater frequency of calling 911 and taking aspirin in response to AMI symptoms (39 times) than the control group (10 times) (p < .001). The response to AMI symptoms in EMT-delivery (46.6%) was more than in direct mail (29.7%) (p < .001)	1.c
Meischke et al.^27^	Multi-forms - Face to Face/Acronym/Teaching - Advertisement - Direct Mail - Telephone-based screening: TBS - Not registering the protocol	6 months after having intervention and 1 time/Data collection will continue until late fall of 2003	No	No	No heart attack survival kit provided	Prospective outcome measurement in late fall of 2003	Intervention176 vs. control 147	The intervention group reported a slightly greater frequency of the coping response to AMI (50%) than in the control group (42%, p < 0.08). The participants who received the kit (n = 107) were more likely to call 911 and take aspirin as a first or second response to AMI (61%) than people who did not receive the kit (39%, p < 0.001). However, a significant difference was found between participants who received the kit from a firefighter (76%) versus those who received the kit on the doorknob (51%, p < 0.001)	1.c
Lefler et al.^28^	- Tailored educational/counseling - Not registering the protocol	60 min	Yes	Yes 15$ Grocery gift card	Attending the Act In Time (modified presentation)	PBQ, and MAPMISS baseline and 1 month after intervention	Intervention group = 48 and Control group = 48	The educational/counseling intervention successfully improved risk identification (p = .004) and accuracy of personal risk beliefs (p < .001) with little change in the control group at one month. The intervention also improved knowledge of MI symptoms (p < .001)	2.d
Meischke et al.^29^	- Face to Face/Acronym/Teaching - Not registering the protocol	6 months	Yes	Yes, The $3.50 paid per house call is an incentive and compensation for gas and other expenses	Never received a Heart Attack Survival Kit (HASK)	Obtained from the MIRFs that are collected by emergency medical services personnel for 1 and 2 years after the intervention	A total of 3,899 chest pain calls were made, 2,026 in intervention areas and 1,873 in control areas	The intervention group had 93 more calls than the control group. This difference was statistically significant (p = 0.04, 95% CI = 4 to 182). Based on the Poisson regression, the intervention group had 1.16 times as many calls as the control group, which also was statistically significant (p = 0.04, 95% CI = 1.03 to 1.30). There were 216 listed calls in the intervention group, compared with 163 in the control group, for a difference of 53 calls and a ratio of 1.32. The percentage of calls with aspirin was 33.9% in the intervention group versus 29.7% of all calls in the control group, a difference of 4.2 percentage points and a ratio of 1.14	1.c
Tullmann et al.^30^	- Tailored educational/counseling - Not registering the protocol	1 and 2 months were to reinforce the learning. The intervention was a one-to-one session with the participant and lasted 30 to 60 min	Yes	No	Not receive any interventions	Data were re-collected at 3 months ( 1 month after intervention)	The sample consisted of 115 adults, 65 years of age or older. Control group n = 57; Experimental group n = 58	Mean difference: Knowledge 2.72 (99% CI: 1.31–4.13), Beliefs 1.88 (0.4–3.37), Perceived control 4.01 (0.73–7.29), Attitudes 0.9 (− 0.17 to 1.98), and Anxiety − 0.61 (− 2.29 to 1.06). There was a statistically significant increase in knowledge [df(1,112) 25.44, p < .001)] and Beliefs [df(1,112) 11.04, p < .001)] without an increase in anxiety in the intervention group. There was no significant difference in attitudes; however, perceived control was a significant difference [df(1,112) 10.27, p < .001)]	1.c
Kalman et al.^31^	- Face to Face/Acronym/Teaching - Not registering the protocol	The intervention was an hour-and-a-half PowerPoint" presentation called Matters of Your Heart	No	Yes, all participants were given a $20 gift card	Not receive any interventions	Immediately after the presentation, the subjects took the post-test, the same as the pre-test, about 30 min to complete	There were 51 women in total	The difference in knowledge of AMI symptom scores was significant. Pre-test mean and standard deviation were 80.98 (6.61), Post-test 94.35 (5.23), p < .01	2.d
Meisinger et al.^32^	- Face to Face/Acronym/Teaching (home visits and telephone calls) - Prospective registration of the protocol	One year. A first home visit was arranged 7–14 days after discharge and took 60–90 min. Study nurses conducted telephone interviews 3, 6, 9, and 12 months after discharge	Yes	No	The control group received the usual care and a one-year follow-up	In both groups at 3, 6, 9, and 12 months after index hospital discharge	Intervention group: n = from 168 to 161; control group: n = from 172 to 168	During one year, there were 57 unplanned readmissions and five deaths in the intervention group, while the control group had 75 unplanned readmissions and three deaths. Concerning the endpoint, the case management program had no significant effect after one year (Hazard Ratio 1.01, 95% confidence interval 0.72–1.41) (p-value 0.969)	1.c
Kirchberger et al.^33^	- Face to Face/Acronym/Teaching (home visits and telephone calls) - Prospective registration of the protocol	One year. A first intervention started 7–14 days after discharge. The interventions delivered by telephone were four in the first year and two in each of the following two years	Yes	No	Patients assigned to the control group received the usual care	All patients had an assessment and examination in the hospital after one year and a final assessment after 3 years which was performed at home in most cases	Intervention group = 161 and control group = 168	The intervention did not significantly affect the time to first unplanned readmission or death (Hazard Ratio 0.89, 95% confidence interval (CI) 0.67–1.19; p = 0.439). However, patients in the intervention group had a significantly better functional status (mean difference: − 0.24 (− 0.41, 0.07), than in the control group	1.c

**Table 3:** Critical Appraisal of the Selected Randomized Controlled Trials (RCT) (Tufanaru et al.^20^).

Studies/total score	Was true randomization used for the assignment of participants to treatment groups?	Was allocation to treatment groups concealed?	Were treatment groups similar at the baseline?	Were participants blind to treatment assignment?	Were those delivering treatment blind to treatment assignment?	Were outcomes assessors blind to treatment assignment?	Were treatment groups treated identically other than the intervention of interest?	Was follow-up complete and if not, were differences between groups in terms of their follow-up adequately described and analyzed?	Were participants analyzed in the groups to which they were randomized?	Were outcomes measured in the same way for treatment groups?	Were outcomes measured in a reliable way?	Was appropriate statistical analysis used?	Was the trial design appropriate, and were any deviations from the standard RCT design (individual randomization, parallel groups) accounted for in the conduct and analysis of the trial?	Total (13)
Meischke et al.^23^	Y	Y	N	Y	U	U	Y	Y	Y	Y	U	Y	Y	9
Hedges et al.^24^	Y	Y	Y	U	U	U	Y	Y	Y	Y	U	Y	Y	9
Luepker et al.^25^	Y	Y	Y	U	U	U	Y	Y	Y	Y	Y	Y	Y	10
Meischke et al.^26^	Y	U	Y	U	U	U	Y	Y	Y	Y	U	Y	Y	8
Meischke et al.^27^	Y	Y	Y	Y	U	Y	Y	U	Y	Y	U	Y	U	9
Meischke et al.^29^	Y	Y	U	Y	U	U	Y	Y	Y	Y	Y	Y	Y	10
Tullmann et al.^30^	Y	Y	N	U	N	U	Y	Y	Y	Y	Y	Y	Y	9
Meisinger et al.^32^	Y	Y	Y	Y	N	Y	Y	Y	Y	Y	Y	Y	Y	12
Kirchberger et al.^33^	Y	Y	Y	Y	N	Y	Y	Y	Y	Y	Y	N	Y	11

**Table 4:** Critical appraisal of the selected quasi-experimental studies (Tufanaru et al.^19^).

Studies/total score	Is it clear in the study what is the cause and what is the effect (i.e. there is no confusion about which variable comes first)?	Were the participants included in any comparisons similar?	Were the participants included in any comparisons receiving similar treatment/care, other than the exposure or intervention of interest?	Was there a control group?	Were there multiple measurements of the outcome both pre and post-intervention/exposure?	Was follow-up complete and if not, were differences between groups in terms of their follow-up adequately described and analyzed?	Were the outcomes of participants included in any comparisons measured in the same way?	Were outcomes measured in a reliable way?	Was appropriate statistical analysis used?	Total (9)
Lefler et al.^28^	Y	N	N	Y	Y	Y	Y	Y	Y	7
Kalman et al.^31^	Y	N	N	N	Y	Y	Y	Y	Y	6

**Table 5:** Quality assessment results of the selected studies by GRADE guideline (Schünemann et al.^21^).

No	^a^Risk of bias (limitation of study design, confounding factors, missing data, adherence measurement)			^b^Precision (methodology, statistical certainty, amount of information on a certain factor how precisely an object of study is measured)		^c^Directness (the extent to which the people, interventions, and outcome measures are similar to those of interest, confident results come from the direct evidence)		^d^Consistency (relevant measurement application where several items that propose to measure the same general construct produce similar scores, no overlapping and missing, statistical significance)		Certainty of evidence
	Low	Unclear	High	Precise	Imprecise	Direct	Indirect	Consistent	Inconsistent	
Meischke et al.^23^	√			√		√		√		High
Hedges et al.^24^	√			√		√		√		High
Luepker et al.^25^	√			√		√		√		High
Meischke et al.^26^	√				√	√		√		High
Meischke et al.^27^	√			√			√	√		Moderate
Lefler et al.^28^		√		√		√		√		Moderate
Meischke et al.^29^	√			√		√		√		High
Tullmann et al.^30^	√			√		√		√		High
Kalman et al.^31^			√	√		√			√	Low
Meisinger et al.^32^	√			√		√		√		High
Kirchberger et al.^33^	√			√		√			√	Moderate

^a^Risk of bias.

^b^Precision.

^c^Directness.

^d^Consistency.

### Systematic review findings

The interventions found in this review were eight, including direct mail, community-based intervention, multi groups health education, innovative methods of using heart attack survival kits and firefighters, tailored educational/counseling intervention, structured education, and counseling intervention, tricked intervention promoting memory and concern, and nurse-based case management. Types of intervention were grouped, and intervention details and content were provided to guide program development and future study. Moreover, how to deliver and implement interventions was also provided for duplicating in future studies if needed.

#### Direct mail

The first intervention was a direct mail campaign in which a Brochure was mailed every two months. The information brochure provided detailed information on acute myocardial infarction symptoms and treatment in a neutral tone. The emotional message was designed to reduce the emotional response of fear, embarrassment, and bothering that could prevent or delay appropriate health behavior. The suggestion for decision-making was the family member's responsibility, not part of the patient's. Unfortunately, Meischke et al. did not provide details of the information or critical message in the brochure; only the concept and principle were found^23^.

#### Community-based intervention

A community-based intervention program was used to reduce out-of-hospital delays. The education message focused on chest pain and other ischemic symptoms, with action if symptoms persisted for 15 min or more. Multiple media channels were used to deliver the message to the general public physicians. Moreover, nurses, paramedics, and other healthcare providers helped deliver the message to high-risk groups. This health education program sequentially emphasized different themes, including general awareness of AMI symptoms and the need for rapid action, development of a Heart Attack Survival Plan, AMI in women, variability of AMI symptoms, bystander response to heart attacks, and use of 9–1-1 to reinforce the primary message^24^.

#### Multi-component strategies & groups health education

The multi-component strategies and groups of health education started with meeting focus groups of AMI patients, relatives, and health care professionals. This meeting found key points for AMI survival, symptom recognition, and the need to act fast by calling 911. The advice was to call 911 for ambulance transport to the hospital if experiencing symptoms persisted for 15 min or longer. This intervention included four strategies. First community organization, in which health professionals and leaders of their relevant organizations in each community constituted a local advisory group. Second, public education targeted all residents of the intervention communities with an 18-month program that included six themes. There was a general awareness of AMI symptoms and appropriate action, MI survival plan, women and MI, MI symptom recognition, bystander response to MI, and the importance of contacting EMS. Third, professional education included physicians, nurses, rehabilitation staff, ED staff, and ambulance staff. They should deeply understand AMI and prompt response in emergencies. Finally, the physician taught patients with a history of coronary heart disease (CHD) or CHD risk factors^25^.

#### The innovation method of using heart attack survival kit & firefighters

The program under the Heart Attack Survival Kit (HASK) project included a heart attack survival kit, red cardboard containing essential information, and a group discussion. A heart attack survival kit contained the following: eye-catching design and adhesive strips for permanent placement in the home; list of the warning signs of AMI; strong recommendation to call 911; strong recommendation to take an aspirin for chest pain; one 325 mg. uncoated adult aspirin, primary step for cardiopulmonary resuscitation (CPR), and space to write in medications/allergies and essential phone numbers. A red cardboard flyer shaped like a door hanger was provided with essential information about AMI and how to act when experiencing AMI. Finally, group discussion issues around cardiac emergencies with seniors in their communities were set^26,27,29^. This innovation program, delivered face-to-face by local firefighters, is designed to increase the utilization of 911 and self-administration of aspirin for seniors experiencing chest pain.

The step-by-step program started with passive-consent letters mailed to the homes of all eligible people in intervention areas (24,582 homes), indicating that a local firefighter would personally deliver a HASK soon unless the participant indicated on a self-addressed postcard that such a visit was not welcomed; the eligible individuals returned the postcard. Two weeks after the passive-consent letters were mailed, local firefighters delivered the kits to the homes of the remaining seniors in King County, Washington. More than 300 local firefighters were trained face-to-face by the staff of this study to discuss the contents of the HASK and to assess and respond to barriers to calling 911 and taking aspirin for chest pain^29^.

#### Tailored educational/counseling intervention

The Tailored educational/counseling intervention included essential information about the pathophysiology of AMI, Symptoms of AMI, the importance of quick responding, appropriate action in AMI situations, a rehearsal plan, and take-home questions. For the step-by-step intervention, all section was provided: (1) act in time, (2) what is a heart attack?, (3) the importance of rapid treatment of heart attack, (4) the ten most common symptoms of MI, (5) expectation and expected reaction to heart attack, (6) step to survival and rehearsing plan, (7) what factors increase one's risk? (8) take-home questions^28^.

#### Structured education and counseling intervention

The highlights of this intervention were specific details of intervention related to preventive behaviors and delaying treatment in AMI situations and a clear program direction by providing a step-by-step approach. This program included five steps. The first was educational and counseling intervention with details. Second, participants were given educational and counseling intervention, including information about typical and atypical symptoms of AMI, how symptoms may vary, and actions to take in the event of AMI symptoms. An advisory form was given to each participant after the intervention. The form, designed by the NHAAP, listed what the participant may feel if experiencing an evolving AMI, medication instructions, directions to call 911 and ask for an ambulance, and the location of the nearest 24-h emergency department. Participants who received the intervention were asked to place the advisory form in a prominent place in their homes. Third, the participants were asked to repeat the information to ensure comprehension. The fourth was that the intervention was delivered in a one-to-one session with the participant and lasted 30 to 60 min, depending on the participant's interest, questions, and comments. Finally, the participants recruited at the senior center were asked to refrain from discussing the study with others to avoid contamination between groups^30^.

#### Tricked intervention promoting memory and concern

One study applied a trick by using abbreviations with keywords to promote female older adult memory and concern. Because female older adults experienced prodromal and atypical/different symptoms of AMI with others, specific symptoms were provided, and acronyms were developed for easy memories. The deliberate misspelling of FACTSS was highlighted for the women to help them remember the long list of warning signs. Prodromal symptoms were FACTSS, which stood for fatigue, anxiety, chest discomfort, tummy (indigestion), shortness of breath, and sleeping difficulties. The MI symptoms were CURB, which represented chest sensation or pain, unusual fatigue, pain radiating back, jaw, or arm pain, and breathing difficulties. The health education matters of your heart using the acronym FACTSS & CURB was applied among female older adults. The program was delivered in the community to groups of women. A script for the researchers to follow provided consistent information to each group. Immediately after the presentation, the subjects took the post-test, the same as the pre-test^31^.

#### A nurse-based case management

The nurse-based intervention is complex, combining components from case management and disease management. Case management focuses on individual care problems of older adults and facility of care coordination; however, disease management pays attention to identifying problems regarding managing AMI symptoms and providing information and individual education. The case-management intervention consisted of a nurse-based follow-up for one year, including home visits and telephone calls. Key elements of the intervention were to detect problems or risks and to give advice regarding a wide range of aspects of disease management, such as symptom management and medication use. A nurse-based case management program includes three steps. First was the initial session, after giving informed consent (so-called “heart book”), followed by home visits. The home visit was arranged 7 to 14 days after discharge, and telephone calls and telephone interviews (at least every three months) were performed. The study nurse assesses the risk level during the first home visit based on compliance, social network, and comorbidities. The higher the risk level, the more contacts (telephone and home visits) were arranged by the study nurse. Finally, the final assessment after 12 months was conducted^32^. However, another study was a case-management intervention consisting of a nurse-based follow-up for three years. This intervention included home visits and telephone calls. The case-management intervention consisted of a three-year nurse-based follow-up, including home visits and telephone calls, risk management, and symptom management^33^.

### Meta-analysis findings

**Figure 2:**
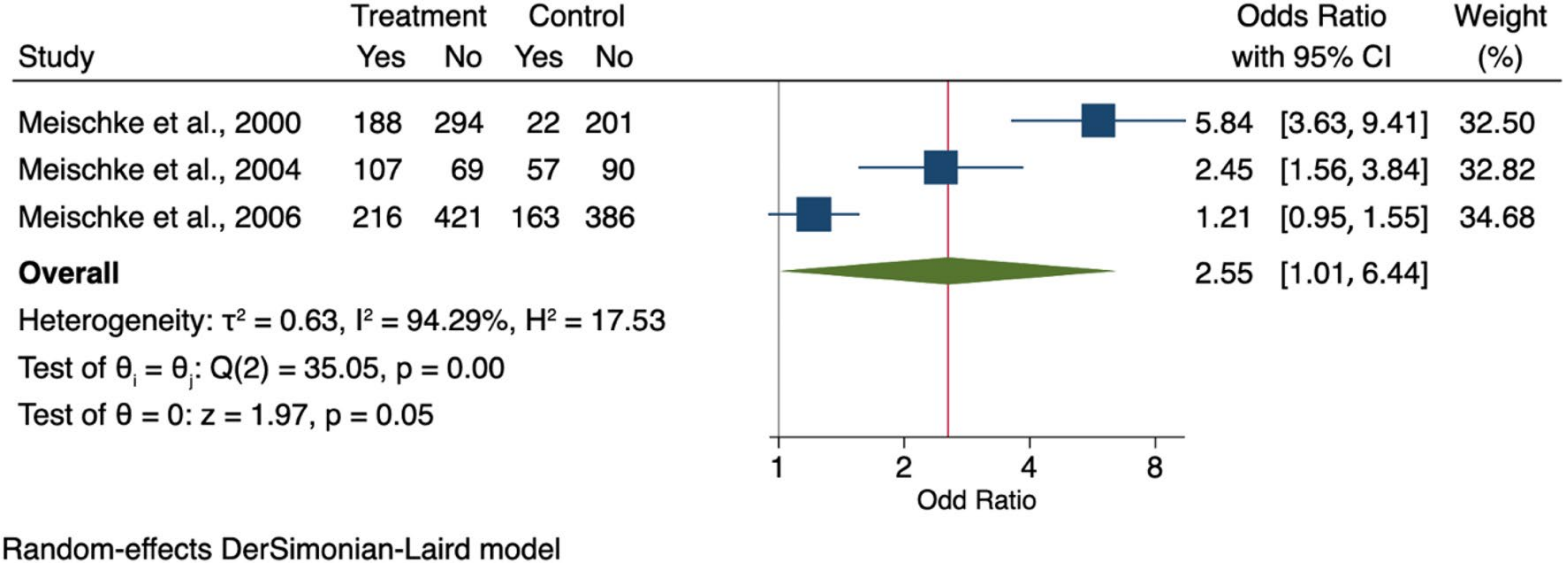
Forest plot for the odd ratios of calling 911 and taking aspirin among AMI patients with and without an intervention: The midpoint of each line illustrates the odds ratio; the horizontal line indicates the confidence interval, and the diamond shows the pooled odds ratio. The red and gray vertical lines indicate the overall effect-size and null-effect values, respectively.

**Figure 3:**
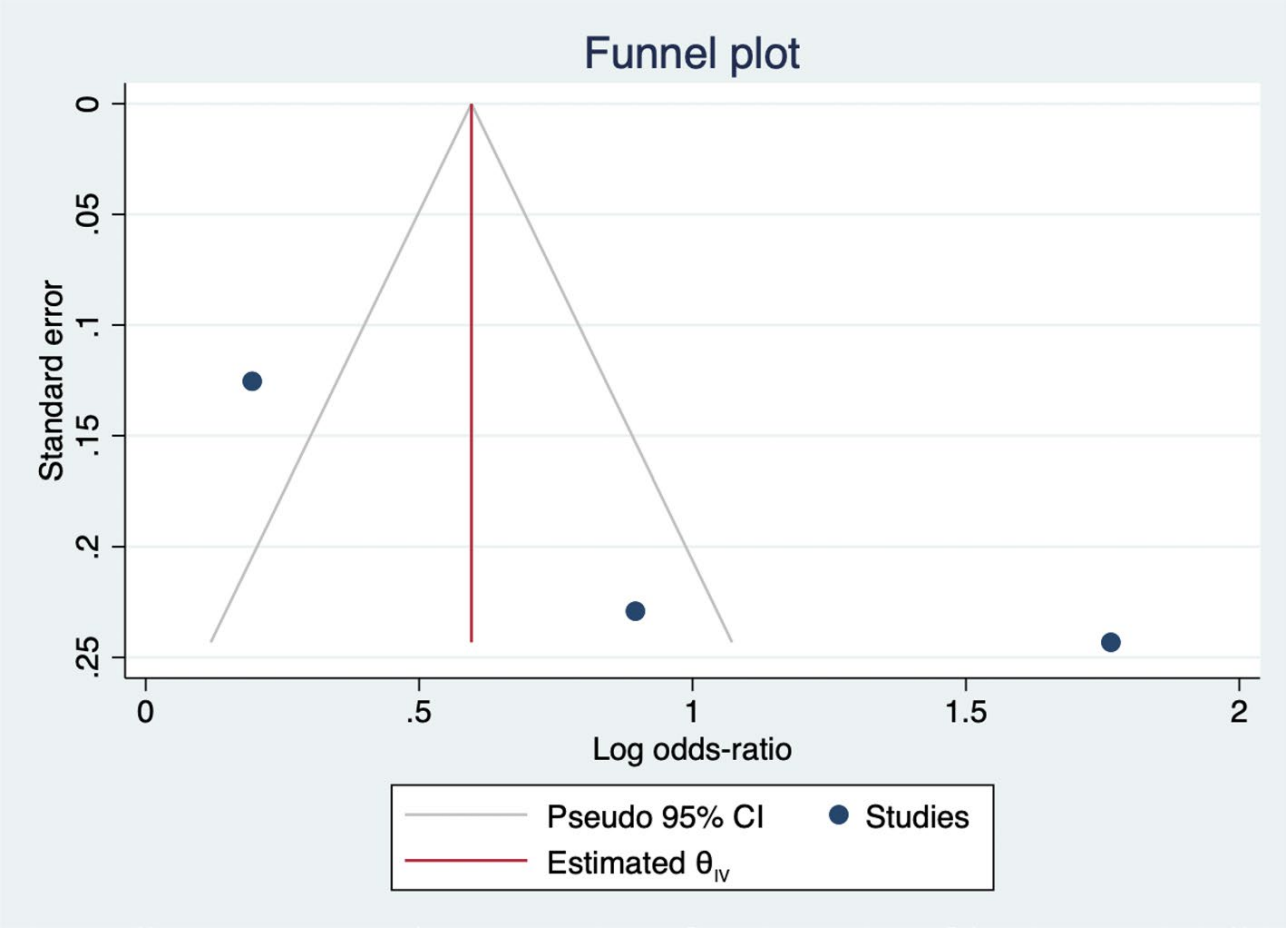
Funnel plot of effect sizes included in the meta-analyses for calling 911 and taking aspirin among AMI patients with and without intervention.

The meta-analysis showed that people receiving a Kit via home by an Emergency Medical Technician or via direct mail or delivered face-to-face by local firefighters had a higher odd of calling 911 and taking aspirin than those without any intervention (OR 2.55, 95% CI 1.01–6.44) as shown in Fig. 2. Publication bias was visually assessed using Begg’s funnel plots and statistically assessed with Egger’s test. Based on publication bias analysis, this figure visually indicates the skewness of the effect sizes observed, as shown in Fig. 3. In addition, the left-sided test for funnel plot asymmetry using Egger’s regression test was significant (p = 0.043), supporting the conclusion that significant publication bias was present.

**Figure 4:**
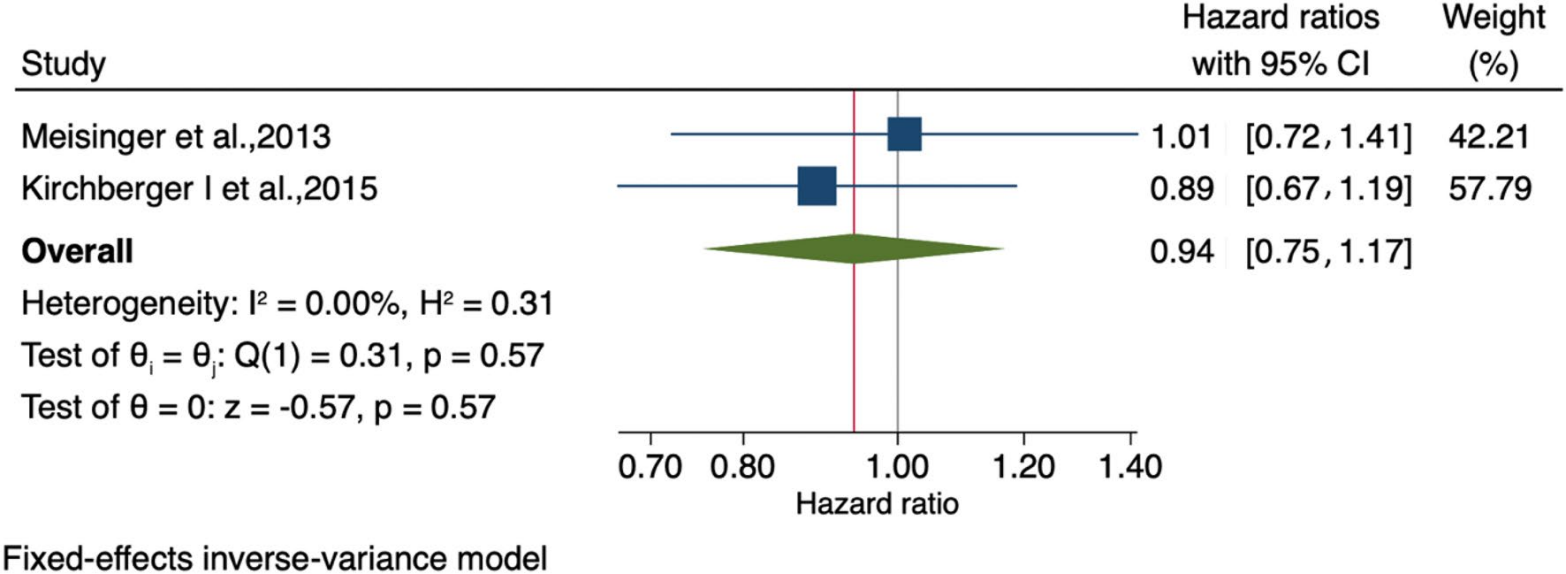
Forest plot for the hazard ratios of affecting time to first unplanned readmission or death among AMI patients with and without an intervention: The midpoint of each line illustrates the hazard ratio; the horizontal line indicates the confidence interval, and the diamond shows the pooled hazard ratio. The red and gray vertical lines indicate the overall effect-size and null-effect values, respectively.

**Figure 5:**
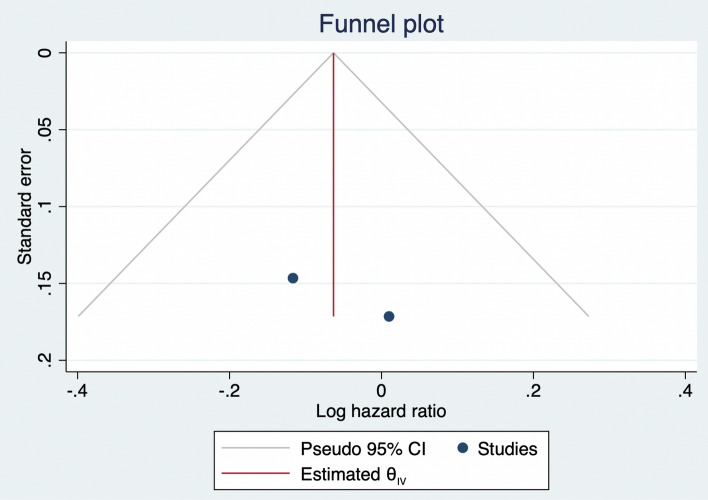
Funnel plot of effect sizes included in the meta-analyses for affecting time to first unplanned readmission or death among AMI patients with and without intervention.

The meta-analysis showed no association between people receiving home-based and nurse-based interventions and those without any intervention regarding time to first unplanned readmission or death (HR 0.94, 95% CI 0.77–1.11), as shown in Fig. 4. Publication bias was visually assessed using Begg’s funnel plots and statistically assessed with Egger’s test. Based on publication bias analysis, this figure provides no visual indication of the skewness of the effect sizes observed, as shown in Fig. 5. The left-sided test for funnel plot asymmetry using Egger’s regression test was non-significant (p = 0.604), supporting the conclusion that non-significant publication bias was present.

**Figure 6:**
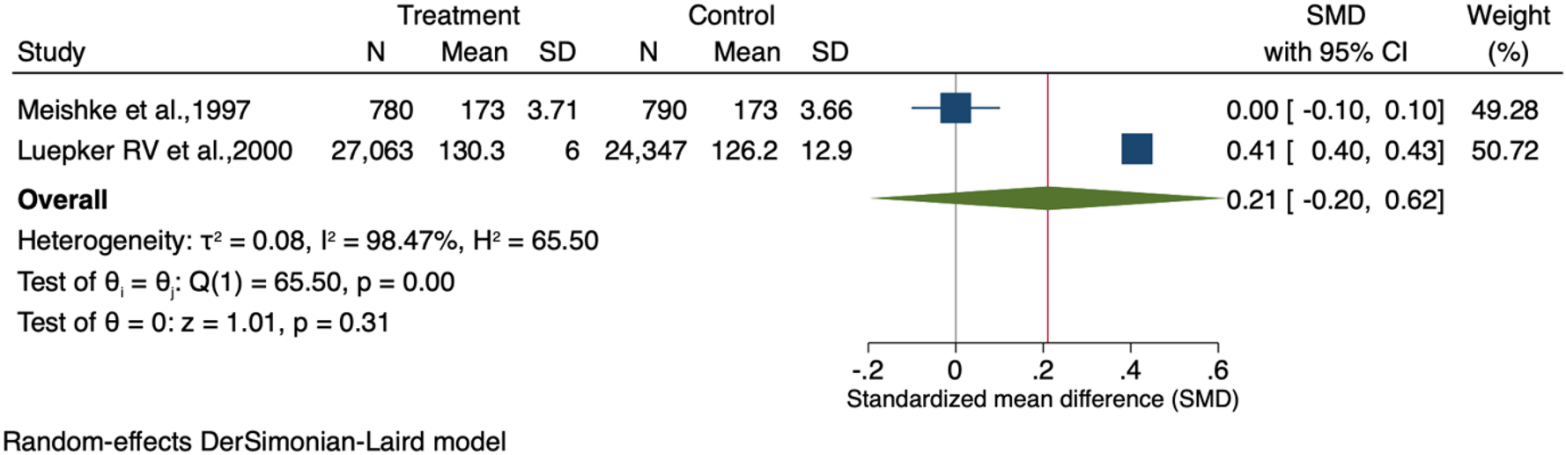
Forest plot for the standardized mean difference of time delay to ER among AMI patients with and without an intervention: The midpoint of each line illustrates the mean difference; the horizontal line indicates the confidence interval, and the diamond shows the pooled mean difference. The red and gray vertical lines indicate the overall effect-size and null-effect values, respectively.

**Figure 7:**
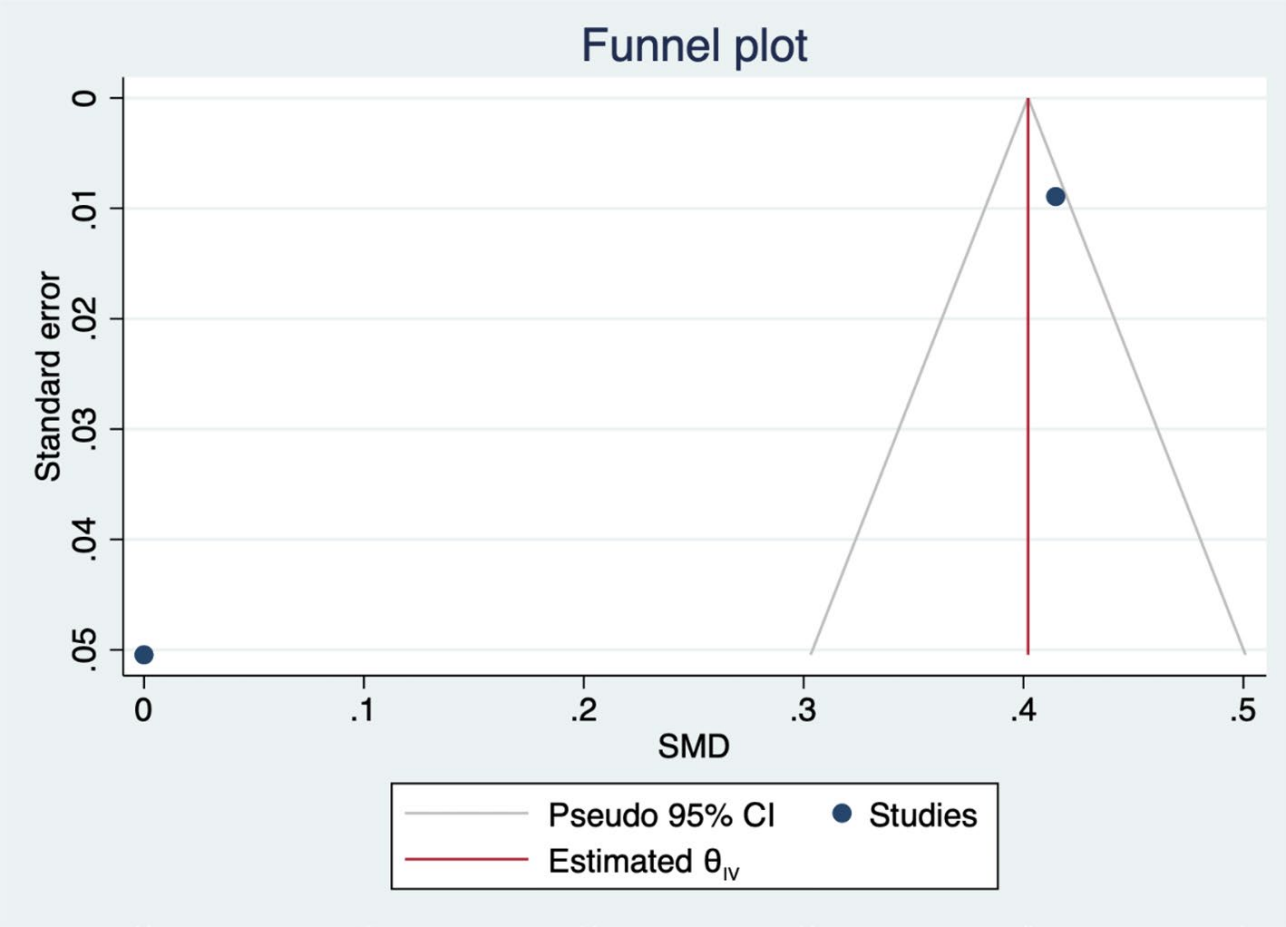
Funnel plot of effect sizes included in the meta-analyses for time delay time to ER among AMI patients with and without intervention.

Finally, the meta-analysis showed no association between people receiving community-based and direct mail intervention and those without any intervention regarding delay time to ER (Standardized mean difference = 0.21, 95% CI − 0.20 to 0.62), as shown in Fig. 6. Publication bias was visually assessed using Begg’s funnel plots and statistically assessed with Egger’s test. Based on publication bias analysis, this figure visually indicates the skewness of the effect sizes observed, as shown in Fig. 7. The left-sided test for funnel plot asymmetry using Egger’s regression test was non-significant (p = 0.441), supporting the conclusion that no significant publication bias was present.

## Discussion

This comprehensive systematic review and meta-analysis synthesized evidence from 11 studies conducted in the USA and Germany focusing on the effectiveness of intervention/health education on health outcomes. Eight of eleven studies were conducted before 2012. It was possible that, in the US and Germany, there are public health policies to call 911 to promote community awareness of emergency health conditions such as AMI and stroke and the need to call 911 since 2000, both at the public and school levels^34,35^. However, the atypical presentation of AMI remains an important issue, especially in older adults and women, to prevent delayed diagnostics and treatments^7,9,28^. Moreover, although morbidity and mortality rates are declining for AMI in most high-income countries, it is rising at an alarming pace for low to middle-income countries^10^. Therefore, AMI intervention for older adults is still needed, mainly conducted on low-middle incomes.

The number of participants in each group ranged from 50 to 226,958 participants. The range is relatively broad because some interventions were conducted as a national community intervention or national campaign^23,25,27^. However, some interventions focused on the individual level^28,30,31^. This systematic review focused on older adults’ studies; most were conducted among women. Older adults and women are areas of concern since they both were the significant groups experiencing atypical presentation, delay in seeking treatment, and result in a high mortality rate^7,9,28^.

The meta-analysis found that people who received any of the three interventions, including receiving a Kit via home by an Emergency Medical Technician, via direct mail, or delivered face-to-face by local firefighters, were more likely to call 911 and take aspirin in an emergency than those who did not receive any intervention. Specifically, the odds of calling 911 and taking aspirin were 2.55 times higher for those who received an intervention, with a 95% confidence interval ranging from 1.01 to 6.44, which is similar to the findings of previous studies^36–38^. However, the study conducted by Mikulík et al. and Fogle et al. observed that the readiness of older adults to call 911 in response to a stroke improved when they were exposed to a video-based educational program^34,39^. In addition, a low-intensity educational campaign, which involved donated advertising media to increase stroke awareness, was unsuccessful. Then, the effectiveness of using different pathways should be explored.

This meta-analysis suggests that providing people with a Kit and information about emergency services can effectively encourage them to seek help in an emergency^26,27,29^. However, it is essential to note that the effectiveness of these interventions may vary depending on the specific context and population being studied. Overall, the findings of this meta-analysis can be helpful for healthcare professionals, emergency responders, and policymakers in designing effective interventions to promote prompt and appropriate responses to emergencies.

Interestingly, many interventions with different pathways were applied to promote knowledge, decision-making, appropriate action, and time to seek treatment; however, based on the meta-analysis, only the odds of calling 911 and taking aspirin were improved. This result might be because the intervention may suit general people but not fit older adults. Older adults own specific characteristics regarding the aging process and their socio-demographic status. First, their aging process and pathogenesis limit older adults’ skills in perceiving information, learning, and understanding compared to younger adults^40,41^. Because the aging process and its pathogenesis limit older adults’ vision, hearing, attention, concentration, and ability to remember information, providing health education or interventions for this group must adhere to the limitations and older adults’ requirements^42,43^. As older adults had reduced memorization ability, the program was designed to have practices to enhance skills and ability to manage the symptoms independently. Moreover, repetitive self-practice would enable practitioners to learn effectively, understand reasons, and achieve sustainable learning^43^. Teaching procedures and periods were not too long. The stimuli transmitted through the five senses, namely, the ears, eyes, nose, tongue, and skin, produce more learning outcomes than stimuli transmitted through one sense only. The practice helped turn the abstract into concrete for better understanding^41,43^.

Second is their socio-demographic status. Older adults have limitations regarding their education, so this condition is an obstacle in accessing, receiving, and understanding health information in taking care of themselves. These include poor reading and listening skills and fear of being scolded by service providers. Their limitations hindered these people from essential and updated health information, so less behavioral modification and appropriate action in illness were found in this age group^44^. Nilnate et al. found that older adults have low to moderate health literacy scores^45^. The limitation of education plus the deterioration of their ears and eyes affect self-care management skills and competency for accessing, understanding, and reporting health knowledge. Therefore, teaching older people should take a short time, be divided into sessions, and consist of explanations and practices. Practices must be simple and uncomplicated in a suitable place, with repeated teaching and training sessions. The teaching style must consider the context and culture of older people while using the proper media, large visible text, simple language, and age-friendly colors to be seen easily and clearly. In addition, teaching materials should be available for older people to take home and review^41–43^. Participation in the teaching and practice process would promote learning, memorization, and practicing with confidence^40,43^. All principles of giving health education to older adults should be considered when developing interventions to solve the delay in seeking treatment in acute myocardial infarction situations.

## Limitations

Four limitations were found in this study. First, some selected outcomes were impossible for meta-analysis; only three variables were included. Second, publication bias was also found in this study; this should be aware when applying results. Third, Egger’s test can lead to misleading results when the number of studies is small; this concern should be considered when interpreting the results. Finally, all studies were from only two countries (USA and Germany); results from this systematic review would limit especially details of intervention from the narrative method because their interventions were developed based on the context of study settings. Then, only the principles should be applied.

## Conclusion and recommendation

Older adult is a significant group experiencing AMI and delaying treatment, causing a high mortality rate. Factors related to their delay differ from other age groups, and their specific characteristics and aging process limit them from recognizing their symptoms and learning new information. This study revealed that the included interventions might not suit and be adequate for older adults to survive the AMI situation because their specific characteristics and aging process would not be a concern when developing intervention. Therefore, specific interventions related to their limitations and needs should be considered when developing interventions for future study and practice.

## Data Availability

The datasets generated and/or analyzed during the current study are not publicly available due to prohibited laws (and/or rules, regulations, and contracts). However, they are available from the corresponding author upon reasonable request.
